# Penalized homophily latent space models for directed scale-free networks

**DOI:** 10.1371/journal.pone.0253873

**Published:** 2021-08-02

**Authors:** Hanxuan Yang, Wei Xiong, Xueliang Zhang, Kai Wang, Maozai Tian

**Affiliations:** 1 School of Statistics, University of International Business and Economics, Beijing, China; 2 Department of Medical Engineering and Technology, Xinjiang Medical University, Urumqi, China; 3 Center for Applied Statistics, School of Statistics, Renmin University of China, Beijing, China; Yunnan University of Finance and Economics, CHINA

## Abstract

Online social networks like Twitter and Facebook are among the most popular sites on the Internet. Most online social networks involve some specific features, including reciprocity, transitivity and degree heterogeneity. Such networks are so called scale-free networks and have drawn lots of attention in research. The aim of this paper is to develop a novel methodology for directed network embedding within the latent space model (LSM) framework. It is known, the link probability between two individuals may increase as the features of each become similar, which is referred to as homophily attributes. To this end, penalized pair-specific attributes, acting as a distance measure, are introduced to provide with more powerful interpretation and improve link prediction accuracy, named penalized homophily latent space models (PHLSM). The proposed models also involve in-degree heterogeneity of directed scale-free networks by embedding with the popularity scales. We also introduce LASSO-based PHLSM to produce an accurate and sparse model for high-dimensional covariates. We make Bayesian inference using MCMC algorithms. The finite sample performance of the proposed models is evaluated by three benchmark simulation datasets and two real data examples. Our methods are competitive and interpretable, they outperform existing approaches for fitting directed networks.

## Introduction

Network analysis is being increasingly prevalent in various scientific disciplines, ranging from anthropology, sociology, social psychology, to physics, mathematics and computer science, among others. Networks provide useful representations for non-Euclidean data and have been employed to analyze interpersonal relationships, academic co-authorships and citations, protein interactions and traffic flows, etc. Among these research, social networks have received excessive discussions, in which nodes typically represent individuals and edges represent social relationships [1–3]. In more general cases, nodes can also be used to denote large social units (for example, families, organizations, governments), objects (airports, servers, locations) or abstract entities (concepts, texts, tasks, random variables), and thus edges indicate the certain relations, states, contents or features of nodes. To date, however, much attention has been paid to model undirected networks.

The aim of this paper is to focus on the directed networks with degree heterogeneity, such as social sharing sites (YouTube, QQzone) and microblogs (Twitter, Weibo). Formally, we use G=(V,E,A) to represent an acyclic directed graph with *n* nodes, where $V={\left\{v_{i}\right\}}_{i=1}^{n}$, $E={\left\{e_{ij}\right\}}_{i,j=1}^{n}$ respectively denotes the sets of nodes and edges, and $\text{A}=\left(a_{1},\ldots ,a_{n}\right)\in R^{n\times p}$ is the attribute matrix of nodes. The topology of a graph can be measured by an adjacency matrix $\text{Y}=\left(y_{ij}\right)\in R^{n\times n}$, where *y*_*ij*_ ∈ {0, 1} indicates the presence or absence of an edge on each ordered pair of nodes (*v*_*i*_, *v*_*j*_), *i*, *j* = 1, …, *n* and *i* ≠ *j*. Edges connecting a node to itself are not allowed, thus *y*_*ii*_ = 0 for *i* = 1, …, *n*. Throughout this paper, we use “*v*_*i*_ → *v*_*j*_” to indicate *y*_*ij*_ = 1.

Many probabilistic models have been proposed in order to capture the topology of graphs by adopting their local properties. The simplest one is the Erdös-Rényi Bernoulli random graph model, in which edges are considered to be independent of each other [4]. Given two arbitrary nodes *v*_*i*_ and *v*_*j*_ in a directed social network, it is more likely for *v*_*i*_ to follow *v*_*j*_ when *v*_*j*_ is following *v*_*i*_, or when both *v*_*i*_ and *v*_*j*_ are connecting to another node *v*_*k*_. In other words, the conditional link probabilities *P*(*y*_*ji*_ = 1|*y*_*ij*_ = 1) and *P*(*y*_*ij*_ = 1|*y*_*ik*_ *y*_*kj*_ = 1) are larger than the marginal link probability *P*(*y*_*ji*_ = 1) [5, 6]. These two properties are called link reciprocity and transitivity. Unfortunately, neither of them is considered in the Erdös-Rényi model. To involve reciprocity, a log-linear statistical model (i.e. *p*_1_ model) is proposed [7] and the stochastic blockmodel is introduced [8], which can also fit the block structure, or network communities by partitioning nodes into different subgroups [9]. The stochastic blockmodel then has a rapid development in various fields [10–12] and is still of great interest in recent research [13–16]. Despite such superiority, the stochastic blockmodels are inappropriate to accommodate the complex dependence structure, such as transitivity, due to the pairwise independence assumption. As a result, the exponential random graph model (EGRM) is proposed as a flexible and alternative way [17–19]. Estimation methods such as the maximum pseudo-likelihood [20] and the maximum likelihood with Markov chain Monte Carlo (MCMC) algorithms [21, 22] are further developed, with a comprehensive comparison conducted in [23].

Another line of network research is the latent space model (LSM), which assumes that each node of a network has a position, denoted as ${\left\{z_{i}\right\}}_{i=1}^{n}\in R^{d}$, in an unobserved latent space [6]. Usually, the dimension of the latent space *d* is small, for example, *d* = 2. To measure the closeness relationship between nodes, the latent positions are involved as latent distances ‖***z***_*i*_ − ***z***_*j*_‖ (could be replaced by any distance). Then the probability of edges *P*(*y*_*ij*_ = 1) is modeled as a function of these positions and node attributes. The above mentioned properties, reciprocity and transitivity, are inherently involved in LSM due to the symmetry of pairwise distances. Handhock et al. introduce the latent position cluster model to involve community structure via multivariate Gaussian mixture model [24], which is further extended to allow for degree heterogeneity by embedding with node-level random effects [25]. Sewell and Chen generalize static model to the dynamic latent space model (DLSM) that accounts for relations drifting over time under the framework of LSM [26]. Such dynamic networks are also studied in [27]. The LSM is widely developed in other directions as well. For instance, Austin propose the covariate-defined latent space random effects model to predict the latent positions of new nodes entering a fitted network [28]. Sewell and Chen develop the model to fit a weighted edges network, which means that the edges connecting nodes are no longer binary variables but can take multi-values [29].

Besides reciprocity and transitivity, degree heterogeneity and homophily attributes are also of great interest in social networks. This work considers all of these properties within the LSM framework. For large-scale social networks, it is reasonable to assume that the degrees of different nodes vary in a wide range. This is also referred as scale-free networks (SFN), in which node degrees follow a power law. Such phenomenon is quite common in online social networks [30]. For example, Facebook, Twitter, LinkedIn and Weibo are popular sites built on social networks, providing communication, storage and social applications for hundreds of millions of users. On these social platforms, it is frequent to see few celebrities capturing substantial numbers of followers, accounting for power law or power law with exponential cutoff degrees. In directed networks, degrees contain in-degrees and out-degrees, defined as $d_{j}^{in}=\sum_{i=1}^{n}y_{ij}$ and $d_{i}^{out}=\sum_{j=1}^{n}y_{ij}$ respectively. The link probability should be strongly related to the heterogeneity of node in-degrees. Taking *v*_*i*_ and *v*_*j*_ as ordinary nodes and $v_{j}^{*}$ as a celebrity with an extremely high $d_{j^{*}}^{in}$, the marginal link probability *P*(*y*_ij*_ = 1) is expected to be much larger than *P*(*y*_*ij*_ = 1). However, it is unlikely for a celebrity to pay close attention to its followers. Thus the conditional link probability *P*(*y*_j*i_ = 1|*y*_ij*_ = 1) should be smaller than *P*(*y*_*ji*_ = 1|*y*_*ij*_ = 1). On the other hand, out-degree heterogeneity only has limit impacts in online social networks, because the number of users one can follow is usually up-bounded (e.g. 5000 in Twitter and 2000 in Weibo), while the total number of nodes in the network is practically countless. As a result, even node *v*_*i*_ keeps a high $d_{i}^{out}$, the link probability for *v*_*i*_ to follow *v*_*j*_ remains around zero. Thus the heterogeneity of out-degrees can be ignored. We call these networks semi-SFN in this paper. Such phenomenon is also discussed in [31], where popularity scaled latent space model (PSLSM) is proposed for large-scale directed network formulation. However, due to the employment of probit function, PSLSM only considers a one-dimensional latent space and limits the latent positions to standard Normal distribution, which is a quite restrictive assumption. To this end, this paper introduces a novel latent space modeling procedure for directed semi-SFN, where the latent distances are scaled by popularity factors ***γ*** = (*γ*_1_, …, *γ*_*n*_) to involve in-degree heterogeneity. The logistic regression extends our proposed model to a much more generalized level. Specifically, the dimension and distribution of latent positions are theoretically unlimited, and homophily attributes are considered emphatically in this paper.

It is well known that the link probability is related to homophily node attributes. Therefore, pair-specific covariates, acting as a distance measure, are introduced in our model. To be mentioned, the classic LSM proposed by [6] also allows for covariates and has been performed in a few research [24, 32]. To the best of our knowledge, however, we are the first to proposed a specific formulation of covariates processing within the LSM framework. In this way, social relationships between nodes can be better represented through latent distances, since the effects of node attributes have been fully extracted. Additionally, to deal with the possible high and ultrahigh dimensionality of covariates, regularization with both ridge and LASSO penalties is discussed under a Bayesian framework, and thus we propose the penalized homophily latent space model (PHLSM). The posterior estimation is performed by adopting MCMC algorithms, which is particularly appropriate in this context since it allows uncertainty of model parameters to be explored through a posterior distribution. Our experiments show that such approach perform well in simulations and real semi-SFN examples compared to other competing models that also involve degree heterogeneity and homophily attributes.

The major contributions of this paper is as follows:

1. We propose a novel latent space model as an alternative network embedding, which comprehensively accommodate the significant properties of directed social networks including reciprocity, transitivity, degree heterogeneity and homophily attributes.
2. The popularity factors are introduced as denominator scales of latent distances so as to model the heterogeneity of node in-degrees in scale-free networks.
3. For different dimensions of covariate spaces, the normal and laplacian priors for regression coefficients are discussed separately as ridge and LASSO regularization within a Bayesian framework.
4. For large-scale online social networks, we randomly sample ego-networks for real data analysis, each of which is formed by a single hub and its followers and keeps the scale-free characteristic. Experimental results demonstrate the superior performance of our approach.

The rest of the paper is organized as follows. A basic description of our proposed models together with a brief illustration in multivariate and high dimensional features are given in the next section. Parameter estimation in Bayesian framework is introduced. Several numeric simulation examples are performed and two real network datasets are fitted. We summarize this work with conclusions.

## Penalized homophily latent space models

We consider a directed network with *n* nodes. Given a *d*-dimensional latent space, a specific position $z_{i}\in R^{d}$, *d* ≥ 1 is allocated to each node. We use $\text{Z}=\left(z_{1},\ldots ,z_{n}\right)\in R^{n\times d}$ to denote the latent position matrix. The data to model consists of a binary adjacency matrix $\text{Y}=\left(y_{ij}\right)\in R^{n\times n}$, where *y*_*ij*_ = 1 if *v*_*i*_ follows *v*_*j*_, *y*_*ij*_ = 0 otherwise, and a pairwise covariate matrix $\text{X}={\left(x_{11},x_{12},\ldots ,x_{nn}\right)}^{\prime }\in R^{n^{2}\times p}$ is derived from a node-specific attribute matrix $\text{A}={\left(a_{1},\ldots ,a_{n}\right)}^{\prime }\in R^{n\times p}$. We then propose two probabilistic models under different dimensions *p*. Note that only binary-valued relations are focused in this paper, though the proposed method can be extended to more complex relational data by transforming the Bernoulli prior of ties.

### PHLSM for multi-covariates

We first discuss the multivariate case, namely *p* ≪ *n*. Assuming edges *y*_*ij*_ to be conditionally independent, the PHLSM is defined as

$$\begin{array}{c} \eta_{ij}=\text{log}\;odds\left(y_{ij}=1|\text{Z},\text{X},\Theta \right)=\beta_{0}+\beta^{\prime }x_{ij}-\frac{\|z_{i}-z_{j}\|}{\gamma_{j}}, \end{array}$$ (1)

where ***β*** = (*β*_1_, …, *β*_*p*_)′ is a *p*-dimensional vector of regression coefficients, ***γ*** = (*γ*_1_, …, *γ*_*n*_)′ is a popularity vector for *n* nodes. Θ = {*β*_0_, ***β***, ***γ***} is the collection of all parameters. Intuitively, as ‖***z***_*i*_ − ***z***_*j*_‖ increasing, the link probability for both *v*_*i*_ → *v*_*j*_ and *v*_*j*_ → *v*_*i*_ will decline. Such symmetric property can accommodate the reciprocity of networks. Throughout this paper, we assume that the latent space coordinates **Z** are independently and identically generated from a 2-dimensional multivariate Normal distribution with mean 0 and equi-variance matrix, i.e.

$$\begin{array}{c} z_{i}∼N_{2}\left(0,\sigma^{2}{\text{I}}_{2}\right), \end{array}$$ (2)

where **I**_2_ is an identity matrix. Moreover, *γ*_*j*_ ∈ (0, 1) is a node-specific popularity scale. The larger *γ*_*j*_, the greater social popularity. Considering extreme cases, if *γ*_*j*_ → 0, the probability for *v*_*i*_ to follow *v*_*j*_ remains 0; When *γ*_*j*_ → 1, we are back to LSM. In this way, the in-degree heterogeneity of semi-SFN can be modeled, meaning that an ordinary node tends to follow a celebrity with high popularity, yet the opposite is not true. For model identification, the intercept *β*_0_ and ∑_*j*_ *γ*_*j*_ is constrained to be 1.

The *p*-dimensional pairwise covariate vectors ***x***_*ij*_ are obtained using an element-wise operator. Specifically, for continuous attributes, the attribute matrix **A** is first normalized columnwisely and then

$$\begin{array}{c} x_{ij}=\left|\text{log}\left(a_{i}\right)-\text{log}\left(a_{j}\right)\right|. \end{array}$$ (3)

For discrete attributes,

$$\begin{array}{c} x_{ij}=\min\{|a_{i}-a_{j}\left|,1\right\}. \end{array}$$ (4)

It is remarkable that attributes play vital roles in our model. In some social network, the probability of a relational tie between two individuals may increase as the characteristics of individuals become more similar. Therefore, in this framework the relative difference between two nodes is of interest. In details, for a continuous attribute normalized in (0, 1), an entropy-like covariate ***x***_*ij*_ is proposed in (3) to measure the relative information diversity. For a discrete attribute, (4) defines a binary covariate ***x***_*ij*_, suggesting that whether nodes *v*_*i*_ and *v*_*j*_ belong to the same category (0 for the same category and 1 otherwise). The purpose for using absolute values of differences is to eliminate the directional factors. In case *p* ≪ *n*, we employ ridge regression coefficients, which equals to the Normal prior for ***β***, i.e.

$$\begin{array}{c} \beta_{k}∼N\left(0,1/\tau_{k}^{2}\right). \end{array}$$ (5)

The feature-specific variance $\tau_{k}^{2}$ serves as a tuning of the *L*_2_ norm penalty within Bayesian framework. Note that when $\tau_{k}^{2}\to 0$, the ridge penalty will degenerate to a non-penalized form, which can lead to an unbiased estimate of *β*_*k*_.

With the implementation of (3) and (4), model (1) has a simple interpretation:

1. For nodes *v*_*i*_ and *v*_*j*_ equidistant from *v*_*k*_, the log odds ratio of *v*_*i*_ → *v*_*k*_ versus *v*_*j*_ → *v*_*k*_ is ***β***′(***x***_*ik*_ − ***x***_*jk*_), that is, the followed probability depends on the similarity of node attributes.
2. For nodes *v*_*i*_ and *v*_*j*_ equidistant from *v*_*k*_, the log odds ratio of *v*_*k*_ → *v*_*i*_ versus *v*_*k*_ → *v*_*j*_ depends on ***β***′(***x***_*ki*_ − ***x***_*kj*_) and $\frac{1}{\gamma_{i}}-\frac{1}{\gamma_{j}}$, thus both attributes and popularity determines the following probability.

### LASSO-based PHLSM for high-dimensional covariates

With the explosion of information, numerous predictors are involved in social network analysis for accurate link prediction, for instance, user preferences in recommender systems, protein connections in protein interactomes and potential communities in social networks. A major challenge in this situation is the high-dimensional regime, where the number of available nodes is typically much smaller than the number of features. It is thus imperative to consider a properly sparse model with low computational complexity.

The log likelihood for (1) is

$$l_{n}\left(\Theta \right)=\text{log}\;P\left(y_{ij}|\eta_{ij}\right)=\sum_{i,j}\eta_{ij}y_{ij}-\text{log}\left(1+e^{\eta_{ij}}\right).$$

To reduce dimensionality, the maximum likelihood estimator with regularization is defined as

$$\begin{array}{c} {\hat{\beta }}^{\lambda_{k}}=\text{arg}\underset{\beta \in R^{p}}{\text{max}}\{l_{n}\left(\Theta \right)-n\sum_{k=1}^{p}p_{\lambda_{k}}\left(\beta_{k}\right)\}, \end{array}$$ (6)

where $p_{\lambda_{k}}\left(\cdot \right)$ is some penalty function with tuning parameter λ_*k*_ ≥ 0 to be determined for each *β*_*k*_. In terms of the ridge regression case (5), the penalty function is described as

$$\begin{array}{c} p_{\tau_{k}^{2}}\left(\beta_{k}\right)=\tau_{k}^{2}\beta_{k}^{2}. \end{array}$$

In this section we discuss high-dimensional cases, where the adaptive LASSO penalty (7) is mainly considered due to its simplest expression and nice properties:

$$\begin{array}{ccc} p_{\lambda_{k}}\left(\beta_{k}\right) & = & \lambda_{k}|\frac{\beta_{k}}{|{\hat{\beta }}_{k}^{\left(0\right)}{|}^{m}}|=\lambda_{k}\left|\beta_{k}\right|, \end{array}$$ (7)

$$\begin{array}{ccc} {\hat{\beta }}_{k}^{\left(0\right)} & = & {\hat{\beta }}_{k}^{\sqrt{n}-consistent} \end{array}$$ (8)

Actually other penalties such as SCAD [33] and MCP [34] are all applicable.

This work performs Bayesian estimation. In Bayesian framework, the *L*_1_ norm penalty in (7) was equivalent to a Laplace distribution (also referred to as the double exponential distribution) for parameter *β*_*k*_ [35], namely

$$\begin{array}{c} \beta_{k}∼Lap\left(0,\frac{1}{\lambda_{k}}\right). \end{array}$$ (9)

It is essential in regularized likelihood methods to determine the tuning parameter λ_*k*_ appropriately, which controls the trade-off between the bias and variance in resulting estimators [36, 37]. Selecting an appropriate tuning parameter becomes an important issue, both theoretically and practically. The most common method for choosing the hyperparameter is the cross validation [38]. Unfortunately, it is difficult to be applied in LSM, since the estimated latent coordinate matrix from the training sets is unfeasible for fitting the testing sets. Rather than setting a fixed number, [39] employs hierarchical priors and assumes the tuning parameter to follow a Gamma prior, which is the conjugate prior of exponential distributions. So a Gibbs sampling algorithm can be implemented for Bayesian estimation, as described in the next section. In our model, we simply extend this hierarchical approach to the adaptive LASSO. Specifically, let *f*_⋅_(⋅) denote the probability density functions, the full conditional posterior distribution for λ_*k*_ is given as

$$\begin{array}{ccc} \pi (\lambda_{k}|\text{Y},\text{X},\beta ,\text{Z},\sigma^{2},\gamma ) & \propto & f_{Lap(0,\frac{1}{\lambda_{k}})}\left(\beta_{k}\right)\cdot f_{\Gamma (\xi ,\delta )}\left(\lambda_{k}\right)\\ & \propto & \frac{\lambda_{k}}{2}\text{exp}\left(-\lambda_{k}|\beta_{k}|\right)\frac{\delta^{\xi }}{\Gamma (\xi )}\lambda_{k}^{\xi -1}\text{exp}\left(-\lambda_{k}\delta \right)\\ & \propto & f_{\Gamma (\xi +1,\delta +|\beta_{k}|)}\left(\lambda_{k}\right), \end{array}$$

where *ξ* is the shape parameter and *δ* is the rate parameter of the Gamma distribution.

## Estimation methodology

We employ Bayesian approach to estimate the parameters in (1) using MCMC algorithms. In Bayesian treatment, a prior distribution *π*(Θ) is placed on Θ and what of interest is the posterior distribution *π*(Θ|**Y**) ∝ *π*(**Y**|Θ)*π*(Θ). In this paper, Metropolis-Hastings (MH) within Gibbs algorithm [40] is adopted for posterior sampling.

### Posterior sampling

We set the priors on the parameters as follows:

$$\begin{array}{c} \begin{array}{cc} \sigma^{2} & ∼IG(\nu ,\phi ),\\ \tau_{k}^{2} & ∼Gamma(\xi_{\tau },\delta_{\tau }),\\ \lambda_{k} & ∼Gamma(\xi_{\lambda },\delta_{\lambda }),\\ \gamma & ∼Dirichlet_{n}\left(\alpha \right). \end{array} \end{array}$$

Here *IG* denotes the inverse Gamma distribution. ***α*** = (*α*_1_, *α*_2_, …, *α*_*n*_) is a strictly positive hyperparameter for the Dirichlet prior. For convenience of notation, all the parameters of PHLSM are collected in Ψ_*r*_ = {**Z**, ***β***, ***γ***, *σ*^2^, ***τ***^2^, ***α***, *ν*, *ϕ*, *ξ*_*τ*_, *δ*_*τ*_} and Ψ_*L*_ = {**Z**, ***β***, ***γ***, *σ*^2^, **λ**, ***α***, *ν*, *ϕ*, *ξ*_λ_, *δ*_λ_}.

The hyperparameters are discussed as follows. For the Inverse Gamma prior of *σ*^2^, *ν* and *ϕ* are expected to be small. Besides we have *E*(*σ*^2^) = *ϕ*/(*ν* − 1) for *ν* > 1, which is supposed to approach the sample variance of initial latent positions. Thus we set *ν* = 2 and $\phi =1/2n\sum_{i=1}^{n}{\left\|z_{i}^{\left(0\right)}\right\|}^{2}$, where $z_{i}^{\left(0\right)}$ indicates the initial value of ***z***_*i*_. For the ridge regression version, it can be shown $\text{Var}\left(\beta_{k}\right)=2\delta_{\tau }^{2}/\left(\left(\xi_{\tau }-1\right)\left(\xi_{\tau }-2\right)\right)$ for *δ*_*τ*_ > 0, *ξ*_*τ*_ > 2, meaning that a large *ξ*_*τ*_ as well as a small *δ*_*τ*_ results in low variability for *β*_*k*_ [39]. So is *ξ*_λ_ and *δ*_λ_ for the LASSO version. As a proposal, we set *δ*_*τ*_ = 0.05, *δ*_λ_ = 0.1, *ξ*_*τ*_ = 4, *ξ*_λ_ = 8 for categorical variables and *ξ*_*τ*_ = 2, *ξ*_λ_ = 4 for continuous variables. Last, the Dirichlet prior for ***γ*** is set to be uninformative, thus a flat Dirichlet distribution, given as *Dirichlet*_*n*_(1, …, 1), is proposed.

Practically, the number of MCMC iterations to reach convergence can be greatly reduced by proper initial values of the latent positions and model parameters. Details for selection of initial values are discussed in the next subsection.

Define

$$\begin{array}{c} p_{ij}=P\left(y_{ij}|\text{X},\text{Z},\Theta \right)=\frac{\text{exp}\{y_{ij}(\beta_{0}+\beta^{\prime }x_{ij}-\|z_{i}-z_{j}\|/\gamma_{j})\}}{1+\text{exp}\{\beta_{0}+\beta^{\prime }x_{ij}-\|z_{i}-z_{j}\|/\gamma_{j}\}}, \end{array}$$

the posterior kernels or full conditional distributions of ridge PHLSM parameters are expressed as

$$\begin{array}{ccc} \pi (z_{i}|\text{Y},\text{X},\beta ,\gamma ,\sigma^{2},\cdots ) & \propto & \prod_{j\neq i}^{n}p_{ij}p_{ji}\cdot f_{N_{2}\left(0,\sigma^{2}I_{2}\right)}\left(z_{i}\right), \end{array}$$ (10)

$$\begin{array}{ccc} \sigma^{2}|\text{Z},\nu ,\phi ,\cdots & ∼ & IG(\nu +n,\phi +\frac{1}{2}\sum_{i=1}^{n}\|z_{i}{\|}^{2}), \end{array}$$ (11)

$$\begin{array}{ccc} \pi (\beta_{k}|\text{Y},\text{X},\text{Z},\gamma ,\tau_{k}^{2},\cdots ) & \propto & \prod_{i=1}^{n}\prod_{j\neq i}^{n}p_{ij}\cdot f_{N(0,\tau_{k}^{-2})}\left(\beta_{k}\right), \end{array}$$ (12)

$$\begin{array}{ccc} \tau_{k}^{2}|\beta_{k},\xi_{\tau },\delta_{\tau },\cdots & ∼ & Gamma(\xi_{r}+\frac{1}{2},\delta_{r}+\frac{\beta_{k}^{2}}{2}), \end{array}$$ (13)

$$\begin{array}{ccc} \pi (\gamma |\text{Y},\text{X},\text{Z},\beta ,\alpha ,\cdots ) & \propto & \prod_{j\neq i}^{n}\prod_{i=1}^{n}p_{ij}\cdot f_{Dir_{n}\left(\alpha \right)}\left(\gamma \right), \end{array}$$ (14)

where the notation “…” indicates that the parameters we do not list are independent of the corresponding variable.

Given posterior distributions of model parameters, the MCMC algorithm can be written as follows:

**Algorithm 1: MCMC algorithm for PHLSM**

0. Set initial values of Ψ_*r*_.

1. For *i* = 1, …, *n*, draw ***z***_*i*_ via MH using a random walk proposal.

2. Draw *σ*^2^ via Gibbs sampling from its full conditional distribution (11).

3. For *k* = 1, …, *p*, draw *β*_*k*_ via MH using a Normal random walk proposal.

4. For *k* = 1, …, *p*, draw $\tau_{k}^{2}$ via Gibbs sampling from its full conditional distribution (13).

5. Draw ***γ*** via MH using a Dirichlet proposal.

  Repeat steps 1–5.

As for the adaptive Lasso version (7), using a maximum pseudo likelihood approximation, the posterior distributions for ***β*** and **λ** can be expressed as

$$\begin{array}{ccc} \pi (\beta_{k}|\text{Y},\text{X},\text{Z},\gamma ,\lambda_{k},\cdots ) & \propto & \prod_{i=1}^{n}\prod_{j\neq i}^{n}p_{ij}\cdot f_{Lap(0,\lambda_{k}^{-1})}\left(\beta_{k}\right), \end{array}$$ (15)

$$\begin{array}{ccc} \lambda_{k}|\beta_{k},\xi_{\lambda },\delta_{\lambda }\cdots & ∼ & Gamma(\xi_{\lambda }+1,\delta_{\lambda }+|\beta_{k}|). \end{array}$$ (16)

Other parameters are the same as the ridge penalty version. The MCMC algorithm is given as Algorithm 2:

**Algorithm 2: MCMC algorithm for LASSO-based PHLSM**

0. Set initial values of Ψ_*L*_.

1. For *i* = 1, …, *n*, draw ***z***_*i*_ via MH, using a Normal random walk proposal.

2. Draw *σ*^2^ via Gibbs sampling using the posterior distribution given in (11).

3. For *k* = 1, 2, …, *p*, draw *β*_*k*_ via MH using a Laplace random walk proposal.

4. For *k* = 1, 2, …, *p*, draw λ_*k*_ Gibbs sampling using the posterior distribution in (16).

5. Draw ***γ*** via MH using a Dirichlet proposal.

  Repeat steps 1–5.

As an aside, there are two remarks for the proposed MCMC algorithms.

**Remark 1**. *The posterior of coordinate matrix* **Z** *is not unique due to the invariance property of distances in a two-dimensional Euclidean latent space by rotation, reflection or translation. To deal with this, the Procrustes transformation* [6] *is applied in each step*.

**Remark 2**. *For Algorithm 2, we use the Dirichlet proposal introduced in* [26] *to draw γ*. *Due to the constraint* |*γ*|_1_ = 1, *all components of γ must keep or remove simultaneously during each iteration*. *To accelerate convergence, we set α*^(*t*)^ = *Mγ*^(*t*−1)^ *at t-th iteration, where M is a sufficiently large positive number*.

### Initialization strategies

As mentioned before, the number of iterations for MCMC to reach convergence can be dramatically reduced by setting appropriate initial values of the parameters Ψ_*r*_ or Ψ_*L*_. Below we give some ad hoc initialization strategies.

1. The initial values of latent positions **Z** can be found using the classical multidimensional scaling (MDS) method [41]. Typically, MDS method could transform an *n* × *n* symmetric matrix of association coefficients between individuals into a unique coordinate matrix in Euclidean space via the principal components analysis approach. In practice, we use the geodesic distances in the directed graph, rescaled by 1/*n*, as the input distance matrix. Then the output coordinate matrix can be employed as the initial latent positions after centralization.

2. For *σ*^2^, a reasonable initial value should be the sample variance of $z_{i}^{\left(0\right)}$, given as

$$\begin{array}{c} \sigma^{2(0)}=\frac{1}{2n}\sum_{i=1}^{n}{\left\|z_{i}^{\left(0\right)}\right\|}^{2}, \end{array}$$

where the superscript (0) indicates the initial value.

3. We use the maximum likelihood estimation of the regression coefficients ***β*** as their initial values. Furthermore, the initial values of $\tau_{k}^{2}$ and λ_*k*_ can be simply obtained via Gibbs sampling with $\beta_{k}^{\left(0\right)}$.

4. Typically for an edge *v*_*i*_ → *v*_*j*_, we expect the value of *γ*_*j*_ to be significantly associated with the in-degree of the end node, i.e. $d_{j}^{in}$, hence the initial value for *γ*_*j*_ is proposed as

$$\begin{array}{c} \gamma_{j}^{\left(0\right)}=\frac{1+d_{j}^{in}}{n+\sum_{h=1}^{n}d_{h}^{in}}. \end{array}$$

The added 1 in the molecule is to promise a strictly positive value for $\gamma_{j}^{\left(0\right)}$, and the corresponding *n* in the denominator is to ensure the summation remaining 1.

## Simulation examples

For evaluation, three different benchmark directed networks datasets are considered. In each dataset several nodes are randomly selected as popular hubs to model the heterogeneity of in-degrees in semi-SFN. For each of them we apply the MCMC algorithm proposed in Algorithm 1 and Algorithm 2. The link sparsity and reciprocity of each adjacency matrix is measured using empirical link probabilities given as follows,

$$\begin{array}{ccc} \hat{P}\left(y_{ij}=1\right) & = & \frac{1}{n(n-1)}\sum_{j=1}^{n}\sum_{i=1}^{n}y_{ij}, \end{array}$$

$$\begin{array}{ccc} \hat{P}\left(y_{ij}y_{ji}=1\right) & = & \frac{1}{n(n-1)}\sum_{j=1}^{n}\sum_{i=1}^{n}y_{ij}y_{ji}, \end{array}$$

$$\begin{array}{ccc} \hat{P}\left(y_{ji}=1|y_{ij}=1\right) & = & \sum_{j=1}^{n}\sum_{i=1}^{n}y_{ij}y_{ji}/\sum_{j=1}^{n}\sum_{i=1}^{n}y_{ij}, \end{array}$$

$$\begin{array}{ccc} \hat{P}\left(y_{j^{*}i}=1|y_{ij^{*}}=1\right) & = & \sum_{j^{*}=1}^{m}\sum_{i=1}^{n}y_{ij^{*}}y_{j^{*}i}/\sum_{j^{*}=1}^{m}\sum_{i=1}^{n}y_{ij^{*}}, \end{array}$$

where *v*_j*_ denotes popular hubs with high in-degrees and *m* is the number of them. The first two equations can reflect the global sparsity of a network. And the last two equations reflect the empirical reciprocity between two arbitrary nodes, or from a popular hub to another node, respectively.

### PHLSM with no covariates

In this example, we consider model (17) without attribute effects,

$$\begin{array}{c} \text{log}\;odds\left(y_{ij}=1|\text{Z},\gamma \right)=1-\frac{\|z_{i}-z_{j}\|}{\gamma_{j}}. \end{array}$$ (17)

The top 5 in-degrees are considered as popular hubs. We generate 20 adjacency matrices to characterize directed social networks, each of which contains 500 nodes. For data generation, we set *σ*^2^ = 3 × 10^−4^, ***γ*** ∼ *Dirichlet*(*α*_1_, …, *α*_*n*_), where *α*_*i*_ are drawn from a power-law distribution, given as

$$\begin{array}{c} P\left(\alpha_{i}=l\right)=\frac{l^{-\theta }}{\sum_{i=1}^{n}\alpha_{i}},\;l\geq 1. \end{array}$$ (18)

Larger *θ* means more likely to produce popular nodes. Three different *θ* are considered in this example for comparison, *θ* ∈ {1.7, 2.0, 2.3}. The means and standard deviations (sd) of empirical link probabilities for all simulation networks are given in Table 1.

**Table 1:** Mean (sd) of the empirical link probabilities for the simulation data.

	*θ* = 1.7	*θ* = 2.0	*θ* = 2.3
$\hat{P}\left(y_{ij}=1\right)$	0.0131 (0.0067)	0.0162 (0.0049)	0.0164 (0.0027)
$\hat{P}\left(y_{ij}y_{ji}=1\right)$	0.0016 (0.0012)	0.0035 (0.0016)	0.0053 (0.0013)
$\hat{P}\left(y_{ji}=1|y_{ij}=1\right)$	0.1028 (0.0424)	0.2053 (0.0569)	0.3196 (0.0561)
$\hat{P}\left(y_{j^{*}i}=1|y_{ij^{*}}=1\right)$	0.0375 (0.0115)	0.0528 (0.0122)	0.0692 (0.0168)

It is shown in Table 1 that the first two empirical probabilities are close to 0. Conversely, the empirical reciprocity conditional probability between arbitrary nodes is much larger, while for an edge sent by a popular hub, the conditional probability remains small.

**Fig 1:**
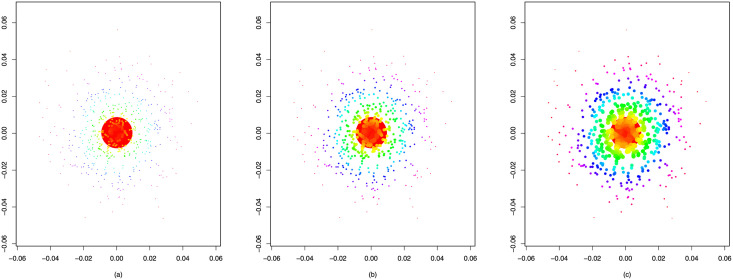
Latent positions scaled by a power-law *γ* with different *θ*. The radius of a circle indicates the value of *γ*_*i*_ for the corresponding latent position. (a) *θ* = 1.7; (b) *θ* = 2.0; (c) *θ* = 2.3.

Fig 1 also presents the latent positions scaled by node popularity, which follows a power-law distribution (18). We can see that with *θ* increasing, the node popularity differences gradually decrease. For *θ* = 1.7, an enormous circle appears near the origin, while the other circles seem to be relatively similar in size, much smaller than the hub. As for *θ* = 2.0 and 2.3, a growing number of moderate-sized circles emerge.

**Fig 2:**
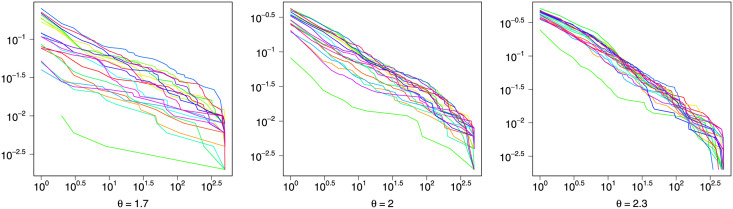
Complementary CDF of in-degrees with different *θ*.

To investigate the power-law of in-degrees, the logarithmic in-degree distribution curves of all simulation networks are depicted in Fig 2. As expected, the empirical logarithmic distribution curves are approximated linear, indicating that the in-degrees follow a power-law, especially when *θ* is relatively large. Note that here we employ the complementary cumulative distribution function (CDF) rather than the probability density function (PDF) because it is more robust against fluctuations resulted from finite sample sizes [42].

To examine the efficiency and accuracy of our proposed methods, we adopt Algorithm 1 to estimate model (17) and set *M* = 5 × 10^6^. Other hyperparameters and initial values are set as described above. We iterate 15,000 times for initial burn-in and another 50,000 times for monitoring. In each iteration, the Procrustes transformation is performed as described in Remark 1. Posterior means of estimates with its standard deviations over 20 simulations are shown in Table 2. It seems that the proposed model performs better for fitting a light-tailed directed semi-SFN.

**Table 2:** Parameter estimates for the no covariate example.

	*θ* = 1.7	*θ* = 2.0	*θ* = 2.3
${\hat{\sigma }}^{2}$	2.989 (0.204)	3.198 (0.264)	4.053 (0.197)
$\text{MSE}(\|{\hat{z}}_{i}-{\hat{z}}_{j}\|)$	1.274 (0.398)	2.009 (1.079)	4.494 (1.225)
$\text{MSE}(\hat{\gamma })$	0.5037 (3.529)	0.1378 (0.939)	0.0459 (0.260)

${\hat{\sigma }}^{2}$ is enlarged 10^4^ times, the MSE of $\|{\hat{z}}_{i}-{\hat{z}}_{j}\|$ and $\hat{\gamma }$ are enlarged 10^3^ times.

**Fig 3:**
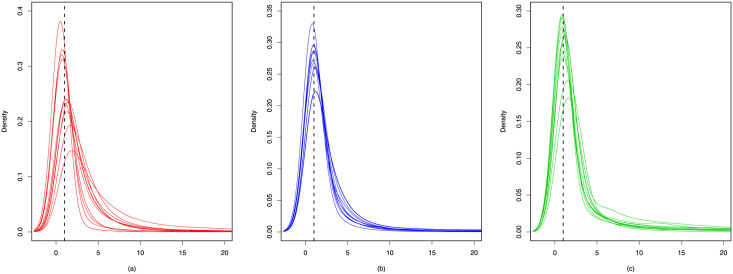
Density curves of the quotients between estimated and true latent distances. (a) *θ* = 1.7; (b) *θ* = 2.0; (c) *θ* = 2.3.

**Fig 4:**
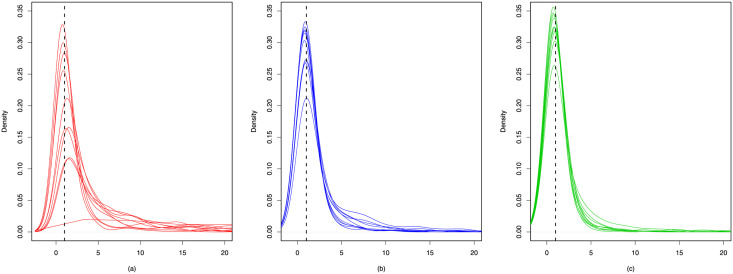
Density curves of the quotients between estimated and true popularity scales. (a) *θ* = 1.7; (b) *θ* = 2.0; (c) *θ* = 2.3.

**Fig 5:**
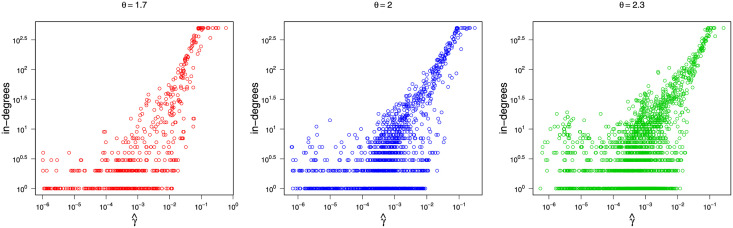
Scatter plots of the estimated popularity and true in-degrees.

We use the following two ratios to compare between the estimates and the truth. For any edge *v*_*i*_ → *v*_*j*_, define $\|{\hat{z}}_{i}-{\hat{z}}_{j}\left\|/\right\|z_{i}-z_{j}\|$ and ${\hat{\gamma }}_{j}/\gamma_{j}$. For each ratio, we depict the density curves of 20 simulation data in Figs 3 and 4. From these two figures we can observe the ratios all concentrate near 1, indicating the superiority of our proposed methods. Furthermore, the trace plots of the estimated popularity and true in-degrees are presented in Fig 5, which show significant positive correlations. Such results empirically verify that the degree heterogeneity and other node-specific random effects can be modeled by rescaling latent distances.

For a careful measurement, total correct rate (TCR), true positive rate (TPR), false positive rate (FPR), and AUC (the area under ROC) are applied to evaluate prediction accuracy. Results are reported in Table 3, which suggests our proposed method performs better with smaller *θ*.

**Table 3:** Mean (sd) of predictive results for the no covariate example.

	*θ* = 1.7	*θ* = 2.0	*θ* = 2.3
TCR	0.972 (0.031)	0.970 (0.016)	0.957 (0.020)
TPR	0.856 (0.086)	0.854 (0.060)	0.831 (0.051)
FPR	0.026 (0.032)	0.028 (0.016)	0.041 (0.020)
AUC	0.915 (0.037)	0.913 (0.025)	0.895 (0.020)

Finally, to examine the dependence of MCMC algorithm on initial values, we take *θ* = 2.0 as a trial. We use uninformative priors for all the parameters. Specifically, initial values of **Z** and ***γ*** are randomly selected from a standard Normal distribution and a flat Dirichlet distribution. The mean(sd) of ${\hat{\sigma }}^{2}$ is 3.019 × 10^−4^(0.247 × 10^−4^), and the AUC value is 0.896(0.036), which is pretty close to the results in Tables 2 and 3 with informative priors. Thus the MCMC algorithm performs robust to the initial values, however it will take longer time to reach convergence.

### PHLSM with multi-covariates

In this example, two attributes ***a***_1_, ***a***_2_ are considered to analyze the node attribute effects. The model for simulation data generation is specified as

$$\begin{array}{c} \text{log}\;odds\left(y_{ij}=1|\text{Z},\text{X},\Theta \right)=1+\beta_{1}x_{ij,1}+\beta_{2}x_{ij,2}-\frac{\|z_{i}-z_{j}\|}{\gamma_{j}}, \end{array}$$ (19)

where *β*_1_ = 0.5, *β*_2_ = −1. ***a***_1_ and ***a***_2_ are assumed to be continuous and binary, generated from a Normal and a Bernoulli distribution respectively, i.e. ***a***_1_ ∼ *N*(0, 1) and ***a***_2_ ∼ *B*(1, 0.5). Thus by the proposed transformation (3) and (4), we obtain *x*_*ij*,1_ and *x*_*ij*,2_. For parameter estimation, 20 simulation datasets are generated. In each replication, we set *θ* = 2, *σ*^2^ = 3 × 10^−4^ as in example 1. Hyperparameters and initial values for implementing Algorithm 1 are set as discussed before. Experimental results are reported in Table 4.

**Table 4:** Bias (sd) of parameter estimates for the multi-covariate example.

	*β*_1_(= 0.5)	*β*_2_(= −1.0)	*σ*^2^(= 3 × 10^−4^)
Bias(sd)	0.001(0.011)	-0.002(0.025)	0.231 × 10^−4^(0.223 × 10^−4^)

From Table 4, we can observe that the proposed MCMC algorithm had a good performance in parameter estimation. The posterior means of ${\hat{\beta }}_{1},{\hat{\beta }}_{2}$ and ${\hat{\sigma }}^{2}$ get very close to true values with quite small standard deviations. In addition, the means (sd) of MSE for $\|{\hat{z}}_{i}-{\hat{z}}_{j}\|$ and ${\hat{\gamma }}_{j}$ are 9.845 × 10^−5^(3.111 × 10^−6^) and 5.165 × 10^−3^ (1.286 × 10^−4^) respectively. The means (sd) of link prediction accuracy are *TCR* = 0.972(0.007), *TPR* = 0.845(0.020), *FPR* = 0.025(0.011), *AUC* = 0.905(0.094). Compared with the predictive results in example 1, it is suggested that the proposed PHLSM can be significantly improved by adding node attributes into the original model.

### LASSO-based PHLSM with high-dimensional covariates

This example focuses on the high-dimensional covariate case. For evaluation and comparison analysis, two groups of simulation experiments are conducted, each of which consists of 20 independent datasets with fixed sample size *n* = 50 and *θ* = 2. All the simulation data come from model (20),

$$\begin{array}{c} \text{log}\;odds\left(y_{ij}=1|\text{Z},\text{X},\Theta \right)=1+\beta^{\prime }x_{ij}-\frac{\|z_{i}-z_{j}\|}{\gamma_{j}}. \end{array}$$ (20)

For the first group, we consider *p* = 40, where ***a***_5_, ***a***_15_, ***a***_25_, ***a***_35_ are significant and the other coefficients are 0. The former 20 attributes are binary and generated from a Bernoulli distribution, i.e. $a_{k}\overset{i.i.d}{∼}B\left(1,0.5\right),k=1,\ldots ,20$. The latter 20 attributes are continuous and generated from a Normal distribution, i.e. $a_{k}\overset{i.i.d}{∼}N\left(0,1\right),k=21,\ldots ,40$. In the second group we consider a higher-dimensional case by setting *p* = 150 and all attributes are produced the same way as in the first group, that is, half of them are binary and the others are continuous, each of which contains 7 significant attributes.

Due to the sparse as well as high dimensional setting, the proposed Algorithm 2 is applied here for posterior estimation with 15,000 iterations for initial burn-in and 50,000 iterations for monitoring. Hyperparameters and initial values are selected as before. As comparison, we also employ Algorithm 1 to fit the simulation data. To investigate the performance of LASSO-based PHLSM on variable selection, we use C to denote the number of non-zero coefficients correctly estimated as non-zero, and IC to denote the number of zero coefficients incorrectly estimated as non-zero. Furthermore, the proportion of the 20 simulations excluding non-zero coefficients from the model is denoted as Under-fit, the proportion of including zero coefficients is denoted as Over-fit, and the proportion for correct coefficient selection is denoted as Correct-fit [43]. Results are presented in Table 5. As expected, the LASSO version results in Table 5 show considerable advantages on fitting a sparse model, especially when *p* is large. Besides, when considering the prediction accuracy, both models have the similar behaviors, between which, however, the LASSO version performs slightly worse. But actually, it is worthwhile to establish a simpler and more interpretable model via sacrificing a little prediction accuracy.

**Table 5:** Results for the high-dimensional covariate example.

	*n* = 50, *p* = 40		*n* = 50, *p* = 150	
	LASSO PHLSM	ridge PHLSM	LASSO PHLSM	ridge PHLSM
C	3.85	4.00	12.60	14.00
IC	0.10	26.75	3.25	119.5
Under-fit	0.10	0.00	0.70	0.0
Over-fit	0.05	1.00	0.45	1.00
Correct-fit	0.85	0.00	0.15	0.00
TCR	0.925 (0.030)	0.941 (0.035)	0.952 (0.023)	0.967 (0.033)
TPR	0.908 (0.116)	0.920 (0.121)	0.948 (0.102)	0.962 (0.078)
FPR	0.067 (0.034)	0.051 (0.027)	0.047 (0.024)	0.031 (0.020)
AUC	0.920 (0.052)	0.935 (0.062)	0.951 (0.045)	0.966 (0.039)

## Real data analysis

For model evaluation, we fit the proposed models in two real data examples. In the first example, we mainly discuss the multi-covariate situation and employ the ridge PHLSM for node representation and link prediction. To compare our model to the state-of-the-art methods, we also consider DLSM, a network model which also considers degree heterogeneity within the LSM framework. The second example focuses on the high-dimensional covariate case. Both regularization versions are fitted to evaluate the feature screening performance of different penalties. We also appropriately modify the proposed models by extending the Normal prior of latent positions to a mixture Normal distribution so as to accommodate the community structure of the network data.

### Pokec data

Pokec is the biggest and most popular Twitter-type online social network in Slovakia. It has connected more than 1.6 million users and the craze has been continuing even after the emergence of Facebook. An in-depth understanding of Pokec is necessary to evaluate current systems, and to understand the impact of social networks on the Internet. The dominant users in Pokec are ordinary individuals, and there also exists some official accounts of governments, enterprises, media, and other celebrities. It provides a platform for individuals to extend and maintain social relationships with others sharing similar interests, and for institutions to make announcements and put advertisements to the public. The raw data extracted by [44] contains the profiles of 1,632,803 users and 30,622,564 directed binary relationships of the whole platform. By using *y*_*ij*_ = 1 to represent the status of user *v*_*i*_ following user *v*_*j*_, we can estimate the empirical probability $\hat{P}\left(y_{ij}=1\right)=1.149\times 10^{-5}$, thus the directed network is extremely sparse. In addition, the maximum of out-degrees is only 8,763, and that of in-degrees achieves 13,733. Actually, most of the hubs with huge amounts of followers are official accounts of media or companies which conduct propaganda through the network.

**Fig 6:**
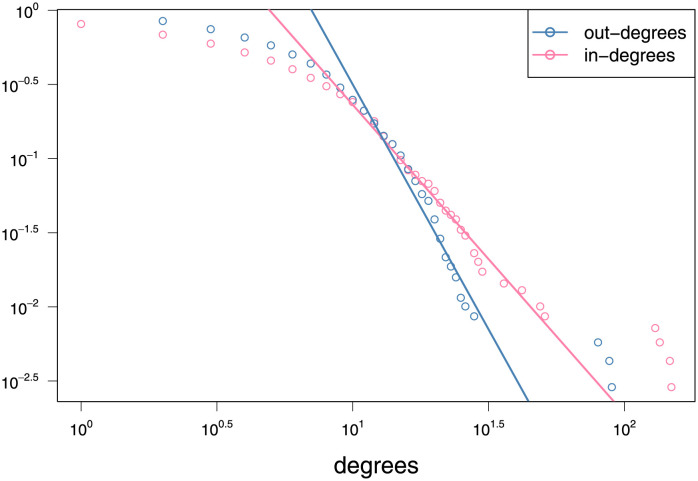
Complementary CDF of node degrees for the Pokec sample network. The solid lines are fitted by scatters excluding non-linear parts at the heads and outliers at the tails.

To adapt this network to the proposed PHLSM model, we draw a sample by randomly selecting 5 popular users and establish a subnetwork using their followees. After eliminating nodes with missing attributes, the final sample size of our subnetwork is *n* = 695. The logarithmic complementary CDF of node degrees are presented in Fig 6. The outliers in tails correspond to the popular hubs selected for the sample network (two of them have the same in-degrees). As can be observed, although both degrees are approximately power-law (ignoring the non-linear head), the range of in-degrees is actually larger than that of out-degrees. The absolute slope of the linear part, namely the exponent *θ* in (18), is steeper for out-degrees than in-degrees. That means the tail of in-degree distribution is fatter and exists more users with either extremely small or extremely large in-degree. Furthermore, the empirical link probabilities of this subnetwork are $\hat{P}\left(y_{ij}=1\right)=0.0106$ and $\hat{P}\left(y_{ij}y_{ji}=1\right)=0.0077$, indicating the sparsity of network. Taking *v*_j*_ as celebrities, the empirical reciprocity conditional probabilities are $\hat{P}\left(y_{ji}=1|y_{ij}=1\right)=0.7293$ and $\hat{P}\left(y_{j^{*}i}=1|y_{ij^{*}}=1\right)=0.3549$. To this end, we regard the subnetwork sample as a semi-SFN and thus employ PHLSM for node representations and link predictions.

The full user profiles of the Pokec data contain 60 user attributes, including user id, gender, region, all friendships public or not, completion percentage of the user file, time the user last logged in, time the user registered, age, and other notes free fillable for users. Due to the severe missing of the user profiles, we only take 4 attributes into our model, namely gender (binary), region (categorical), age (continuous), and registration time (continuous). To be specific, the regions are categorized at state (in Slovakia) or country (out of Slovakia) level, and any sample with zero age are identified as missing and deleted. We then propose to estimate

$$\begin{array}{c} \text{log}\;odds\left(y_{ij}=1|\text{Z},\text{X},\Theta \right)=1+\beta_{1}gender_{ij}+\beta_{2}region_{ij}+\beta_{3}age_{ij}+\beta_{4}time_{ij}-\frac{\|z_{i}-z_{j}\|}{\gamma_{j}}, \end{array}$$

where *y*_*ij*_ = 1 if user *v*_*j*_ is a friend of *v*_*i*_ (but user *v*_*i*_ is not necessarily to be a friend of *v*_*j*_), Θ = {***β***, ***γ***, *σ*^2^, ***τ***^**2**^} is a collection of parameters. The continuous and discrete attributes are respectively processed according to (3) and (4).

**Fig 7:**
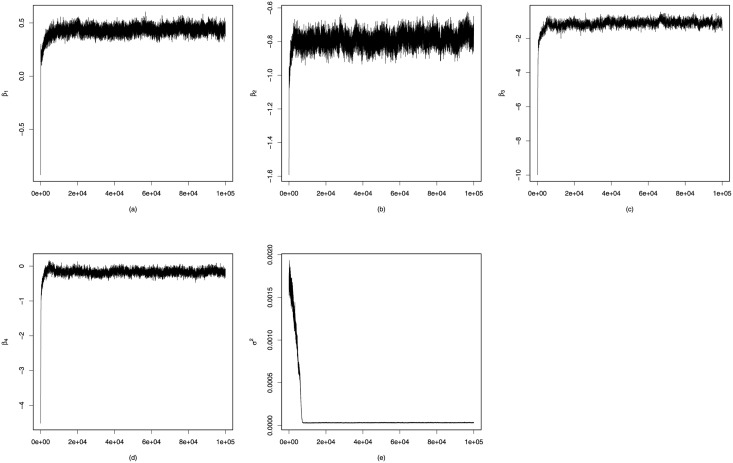
Trace plots for parameters fitting the Pokec subnetwork.

We run 100,000 iterations, including 30,000 for initial burn-in and 70,000 for monitoring. The trace plots for parameters *β* and *σ*^2^ are given in Fig 7. Posterior estimates of parameters are ${\hat{\beta }}_{1}=0.438,{\hat{\beta }}_{2}=-0.786,{\hat{\beta }}_{3}=-1.089,{\hat{\beta }}_{4}=-0.169$. Typically, there should exist homophily relationships, that is, nodes sharing similar attributes are more likely to form ties. In this experiment, results of ${\hat{\beta }}_{2}$, ${\hat{\beta }}_{3}$ and ${\hat{\beta }}_{4}$ suggest that the region, age and registration time are homophily attributes, where the last exerts slight effects. On the other hand, the result of ${\hat{\beta }}_{1}$ indicates that the gender attribute presents heterophily characteristic, which means in an average sense users with different genders tend to be more intimate. It is reasonable for such results since people are usually more interested in the opposite sex during social activities. Nevertheless, those from vicinal regions or with similar ages are more probable to share common topics and become friends.

**Fig 8:**
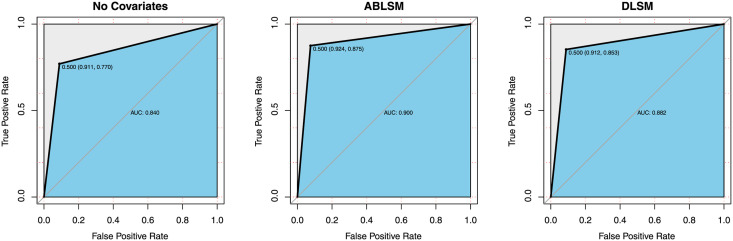
ROC curves for models fitting the Pokec subnetwork.

Our models (with and without covariates) are compared to DLSM proposed by [26]. Specifically, we simplify the dynamic approach to fit a static network by ignoring the time *t* for each latent position, and the covariates are involved in the same way as in PHLSM, given as

$$\begin{array}{ccc} \text{log}\;odds(y_{ij}=1|\text{Z},\text{X},\Theta ) & = & \beta_{1}gender_{ij}+\beta_{2}region_{ij}+\beta_{3}age_{ij}+\beta_{4}time_{ij}\\ & & +\beta_{in}\left(1-\frac{\|z_{i}-z_{j}\|}{r_{j}}\right)+\beta_{out}\left(1-\frac{\|z_{i}-z_{j}\|}{r_{i}}\right), \end{array}$$

where Θ = (***β***, *β*_*in*_, *β*_*out*_, ***r***) and ***r*** = (*r*_1_, …, *r*_*n*_) is a node-specific influence factor. Experimental results are reported in Table 6. ROC curves of the three models are depicted in Fig 8. Intuitively, introducing the node attributes can dramatically improve prediction accuracy, such as TPR and AUC, and our model performs better than DLSM for fitting the semi-SFN over all of the four predictive indices. For inference, PHLSM iterates less than 10,000 times for the Markov chain to reach convergence, as is shown in the trace plots (see in Fig 7), while DLSM iterates more than 60,000 times for convergence. The running time for estimating PHLSM using the MCMC algorithm with 100,000 iterations is 5.48 hours in R on a 2.6 GHz processor, and that for DLSM is 6.34 hours, due to the more parameters to estimate.

**Table 6:** Predictive results for the Pokec subnetwork.

	No covariates	PHLSM	DLSM
TCR	0.909	0.923	0.912
TPR	0.770	0.875	0.853
FPR	0.089	0.076	0.088
AUC	0.840	0.900	0.882

### Twitter ego-network data

Almost everyone encounters hundreds or thousands of people since childhood, but the number of friends that can be keep in touch simultaneously is very limited. Anthropologist Dunbar points out that there is an upper limit to the ability of human beings to maintain social relations, which is about 150 [45]. This upper limit is determined by the physiological characteristics of primates. Recent studies have shown that the upper limit has not been breached because of the higher communication efficiency, such as mobile phones, social networking sites (for instance see [46]). Regarding a person (ego) and his/her friends as nodes and the friendships between this person and his/her friends as edges, we can get an ego-centered network, or more briefly, an ego-network. Ego-networks are very important in anthropology. They are not only helpful for the detailed study of individual characteristics, but also can be extended to the study of the structure and function of social networks.

In this example, we consider 3 sets of ego-network data crawled in Twitter [47], with 28, 10 and 12 users respectively. In each ego-network, the users are in a relatively close relationships due to the small circle size, and the ego is assumed to be followed by every other users in the circle. However, users from different ego-networks are barely connected, giving rise to a classical community structure of social networks. It is inappropriate to apply the original PHLSM here because the egos can only be considered as hubs in their own circles, rather than global hubs. To accommodate our model in such clustering networks, we refer to [24] and assume the latent positions to be drawn from a mixture multivariate Normal distribution, described as

$$\begin{array}{c} z_{i}∼\sum_{g=1}^{G}\delta_{g}N_{2}\left(\mu_{g},\sigma_{g}^{2}{\text{I}}_{2}\right), \end{array}$$ (21)

where *G* is the number of clusters and is 3 in this example, *δ*_*g*_ is the prior probability for node *v*_*i*_ belonging to cluster *g*, and *μ*_*g*_, $\sigma_{g}^{2}$ denote the mean and variance of each cluster. The posterior probability of clustering labels is then given as

$$\begin{array}{c} P\left(k_{i}=g|\text{Z},\delta_{g},\mu_{g},\sigma_{g}^{2},\cdots \right)=\frac{\delta_{g}f_{N_{2}\left(\mu_{g},\sigma_{g}^{2}{\text{I}}_{2}\right)}\left(z_{i}\right)}{\sum_{h=1}^{G}\delta_{h}f_{N_{2}\left(\mu_{h},\sigma_{h}^{2}{\text{I}}_{2}\right)}\left(z_{i}\right)}, \end{array}$$

where *k*_*i*_ denotes the clustering label of node *v*_*i*_. The prior distributions for *δ*_*g*_, *μ*_*g*_, and $\sigma_{g}^{2}$ are chosen as conjugate priors, corresponding to Dirichlet, Normal, and Inverse Gamma distribution respectively.

One more thing to be mentioned is the recognition problem, which is so called the “label switching” problem [48], the mixture model is insensitive to the order of clustering labels, because the likelihood of (21) is the same for all permutations of labels. In this example we post-process the MCMC posterior samples by selecting a permutation of clustering labels to minimize the Kullback-Leibler divergence. See [24] for more details.

The node attributes are the hashtags (#) and mentions (@) extracted from each user’s tweets. In this experiment we totally take 112 attributes into consideration, each of which is a binary feature, representing whether the user’s tweets include a particular hashtag or mention. In practice, it is reasonable to conjecture that most of the features are insignificant, thus a sparse model should be proposed via the LASSO-based PHLSM. To evaluate the feature screening and link prediction of the proposed models, we also fit the ridge PHLSM and obtain a full model, that is, all covariates are retained in the model. Both proposed models are modified by transforming the latent position prior to a mixture Normal distribution to accommodate the community structure.

To estimate PHLSM, we perform the proposed MCMC algorithms with 60,000 iterations, still 10,000 for initial burn-in and 50,000 for monitoring. Finally 7 significant features are selected in the sparse model, listed in Table 7. It seems that Twitter topics of greatest interest are distracted driving, photos and ttot.

**Table 7:** Significant attributes selected for the Twitter data.

content	type	parameter estimates
#distracteddriving	hashtag	-0.7309
#photos	hashtag	4.1559
#ttot	hashtag	1.7596
@Smithsonian	mention	-0.0644
@klout	mention	3.5662
@sesamestreet	mention	-0.0414
@weatherchannel	mention	2.5561

**Fig 9:**
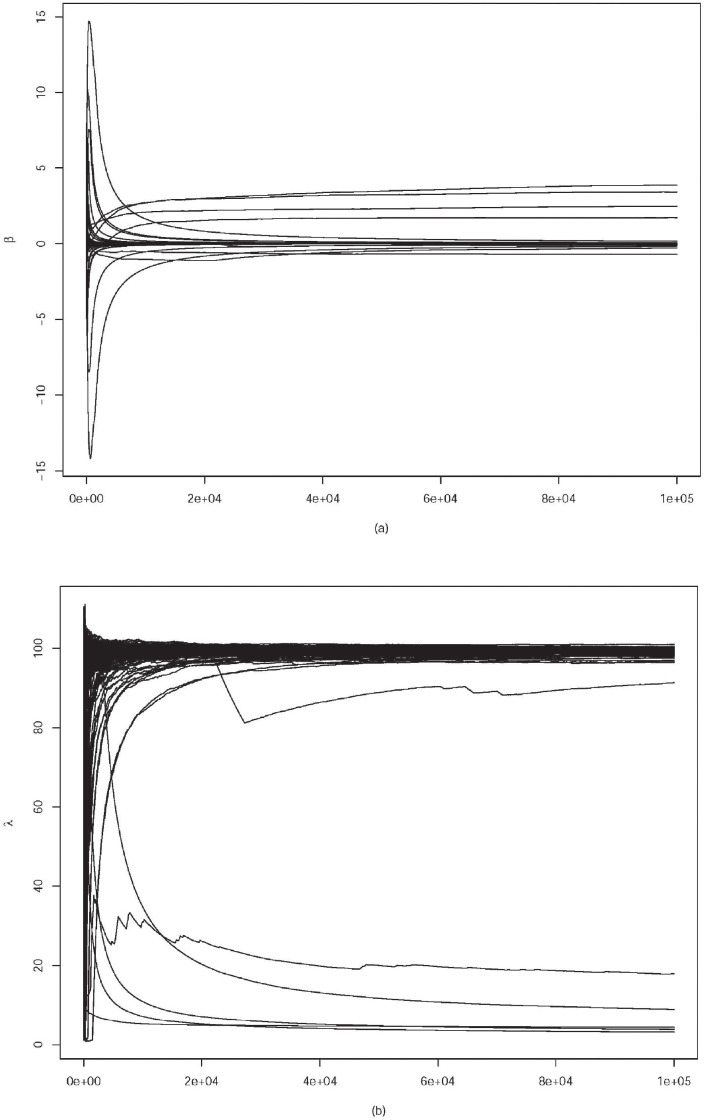
Cumulative mean plots of MCMC posterior samples for model parameters in fitting the Twitter ego-network. (a) Covariate regression coefficients ${\hat{\beta }}_{k}$; (b) Tuning parameters ${\hat{\lambda }}_{k}$.

**Fig 10:**
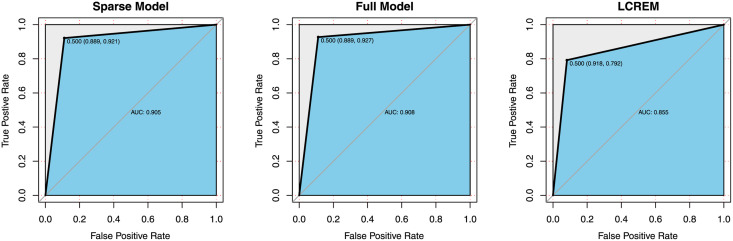
ROC curves for models fitting the Twitter ego-network.

Cumulative mean plots for all regression coefficients and tuning parameters are depicted in Fig 9. Results of link prediction are reported in Table 8. As comparison, we also fitted the latent cluster random effects model (LCREM) proposed by [25], which incorporates the degree heterogeneity by adding node-specific random terms to the log odds. It can be demonstrated that the predictive results are very similar for the two forms of PHLSM, but the sparse model only includes 7 covariates, which is much simpler than the full version with 112 covariates. Such results can reflect superiority of the LASSO method for feature screening. On the other hand, LCREM shows poor performance, especially for predicting the true positive entities. ROC curves of the three models are presented in Fig 10.

**Table 8:** Predictive results for the Twitter ego-network.

	Sparse Model	Full Model	LCREM
TCR	0.8894	0.8894	0.9073
TPR	0.9213	0.9270	0.7921
FPR	0.1107	0.1111	0.0818
AUC	0.9053	0.9079	0.8552

**Fig 11:**
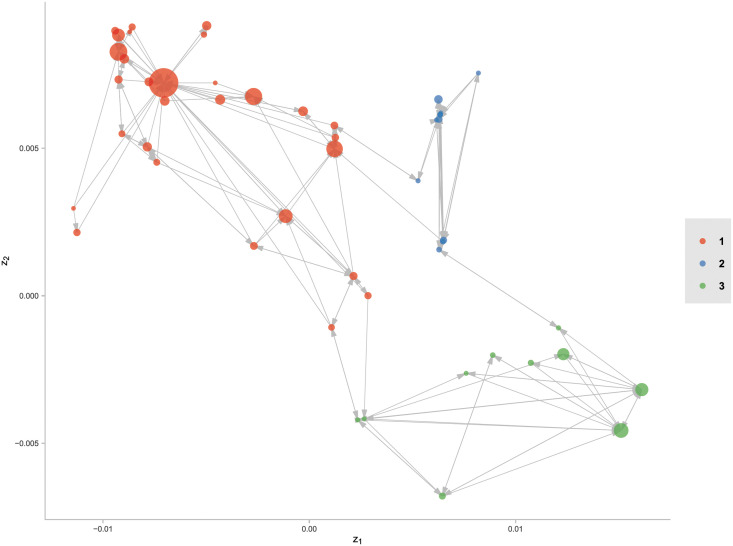
Directed graph with latent positions for the Twitter ego-network. The circles are located based on the estimated latent positions, and the directed edges denote the true relations of users. The colors and sizes of circles denote the true user clustering labels and estimated popularity scales respectively.

Directed graph for the fitted ego-network are reported in Fig 11. The circles are located based on the estimated latent positions, and the directed edges denote the true relations of users. The colors and sizes of circles denote the true user clustering labels and estimated popularity scales respectively. Specifically, most of the popular users, denoted in large sizes, concentrate near the center of a community, while those on borders only have few followers, denoted in small sizes. In addition, the latent positions from different communities are separated clearly, suggesting the importance of community detection in fitting such multi-ego-networks.

## Conclusions

This paper introduces the penalized homophily latent space models for directed social networks. The proposed Bayesian inferential approaches achieve superior performances in fitting two real data examples. Typically, the proposed models accommodate typical network properties, such as reciprocity and transitivity within the LSM framework. The first major innovation of the proposed methods is to improve extensive applicability and predictive accuracy by introducing pairwise node attributes. Besides, the popularity scales are also considered to involve the heterogeneity of node in-degrees. The model performs well for node representation and link prediction for semi-SFN. An alternative approach for network visualization is yielded, which can reflect the social relationships among individuals, as well as their popularity in a social network. For model evaluation, we compare our models with other network modeling frameworks such as DLSM. It appears that our models, with a more concise form and less computation costs, outperform the state-of-the-art approaches.

## Supporting information

S1 Data (7Z)
